# Classical and Bayesian Inference of Conditional Stress-Strength Model under Kumaraswamy Distribution

**DOI:** 10.1155/2021/1087871

**Published:** 2021-07-30

**Authors:** Fathy H. Riad, Mohammad Mehdi Saber, Mehrdad Taghipour, M. M. Abd El-Raouf

**Affiliations:** ^1^Department of Mathematics, College of Science, Jouf University, Sakaka, Saudi Arabia; ^2^Department of Mathematics, Faculty of Science, Minia University, Minia, Egypt; ^3^Department of Statistics, Higher Education Center of Eghlid, Eghlid, Iran; ^4^Department of Statistics, Faculty of Sciences, University of Qom, Qom, Iran; ^5^Basic and Applied Science Institute, Arab Academy for Science, Technology and Maritime Transport (AASTMT), Alexandia, Egypt

## Abstract

Stress-strength models have been frequently studied in recent years. An applicable extension of these models is conditional stress-strength models. The maximum likelihood estimator of conditional stress-strength models, asymptotic distribution of this estimator, and its confidence intervals are obtained for Kumaraswamy distribution. In addition, Bayesian estimation and bootstrap method are applied to the model.

## 1. Introduction

In reliability, *R*=*P*(*X* > *Y*) is named as the “stress-strength model.” This model has application in some literatures in addition to reliability, such as biostatistics, quality control, engineering, psychology, stochastic precedence, medicine, and probabilistic mechanical design. For a comprehensive review with details, refer to the study by Kotz et al. [1]. Regarding this reference, a sample in a clinical study can be considered in such a way that *Y* and *X* are assumed to be the results of a treatment group and a control group, respectively. Therefore, the expression (1 − *R*) measures the effect of treatment.

For some other applications, refer to the study by Ventura and Racugno (2011). In terms of reliability, *Y* is considered the strength of a component, which is under *X* stress. Therefore, *R* and (1 − *R*) indicate the system performance and the probability of system failure, respectively.

Some authors have extensively studied quantity *R* in parametric case for different distributions and in nonparametric case. Refer the study by Rezaei et al. [2] for a list of distributions used in this matter.

In usual situations, it is known that *X* and *Y* are bigger than two fixed values. Especially when *X* and *Y* are lifetime of two components of a system, we may know that these components had been alive for a known time until we are going to have some inferences about *R*=*P*(*X* > *Y*). Therefore, Saber and Khorshidian [3] introduced the conditional stress-strength model as follows:

(1) $$\begin{array}{c} R^{|a,b}=P\left(X>Y|X>a,Y>b\right). \end{array}$$

The quantity *R* is special case of this quantity if we set *a*=*b*=−*∞*. In the study by Saber and Khorshidian [3], if independent random variables *X* and *Y*  are continues, then

(2) $$\begin{array}{c} R^{|a,b}=\left\{\begin{array}{c} \frac{1-F_{Y}\left(a\right)-\int_{a}^{+\infty }F_{X}\left(y\right)f_{Y}\left(y\right)\text{ d}y}{\left(1-F_{X}\left(a\right)\right)\left(1-F_{Y}\left(b\right)\right)};\;a=b,\\ \frac{1-F_{Y}\left(b\right)-\int_{b}^{+\infty }F_{X}\left(y\right)f_{Y}\left(y\right)\text{ d}y}{\left(1-F_{X}\left(a\right)\right)\left(1-F_{Y}\left(b\right)\right)};\;a<b,\\ \frac{\int_{a}^{+\infty }F_{Y}\left(x\right)f_{X}\left(x\right)\text{ d}x-F_{Y}\left(b\right)\left(1-F_{X}\left(a\right)\right)}{\left(1-F_{X}\left(a\right)\right)\left(1-F_{Y}\left(b\right)\right)},\;a>b. \end{array}\right. \end{array}$$

They worked on exponential distribution in the first paper on conditional stress-strength models.

In this paper, the Kumaraswamy distribution is applied to conditional stress-strength models. The Kumaraswamy distribution is a continuous distribution whose values are similar to the beta distribution in the distance [0.1]. In this respect, it is very similar to the beta distribution. However, it has significant advantages in terms of performance, and the cumulative irreversible distribution of booklets has advantages.

In the study by Kumaraswamy [4]; Kumaraswamy [5], hydrological data were shown, for example, daily rainfall and daily flow are not compatible with known and widely used distribution distributions such as normal, log-normal, and beta distributions. In addition, distributions such as Johnson and natural transformed polynomials have this problem too. Therefore, he defined a new probability density function called the sine probability density function. It seems that researchers for such data have considered this type of distribution (see Sundar and Subbiah [6]; Fletcher and Ponnambalam [7]; Seifi et al. [8]; Ponnambalam et al. [9]; and Ganji et al. (2006)).

The Kumaraswamy (Kw) distribution has a probability density function (pdf) and a cumulative distribution function as follows:

(3) $$\begin{array}{c} f_{X}\left(x\right)=\alpha \beta x^{\alpha -1}{\left(1-x^{\alpha }\right)}^{\beta -1},\\ F_{X}\left(x\right)=1-{\left(1-x^{\alpha }\right)}^{\beta }, \end{array}$$

respectively, where *x* ∈ [0,1] and *α*, *β* ∈ (0, *∞*) are two positive shape parameters. Some works on stress-strength models by using of this distribution have been listed as follows. Nadar et al. [10] studied classical and Bayesian estimation of *R*, and then Nadar and Kizilaslan [11] performed the same study but by using upper record values. Estimation of reliability of multicomponent stress-strength models (introduced by Bhattacharyya and Johnson (1974)) has been studied by Dey et al. [12]. Finally, the multicomponent stress-strength model based on progressively censored sample has been worked by Kohansal [13].

The rest of the paper is organized as follows; we devote Section 2 to study *R*^*|a*,  *b*^ in case of Kumaraswamy distribution. In a continuation of Section 2, the ML estimator of quantity *R*^*|a*,  *b*^, its corresponding asymptotic distribution, and confidence interval are presented. Furthermore, two methods including Bayesian and bootstrap are applied to our recommended model. Finally, results are presented in Section 3.

## 2. Conditional Stress-Strength Model for Kumaraswamy Distribution

In this section, quantity (2) is computed when distribution of components is Kumaraswamy.

**Theorem 1 .**

Suppose *X* ~ Ku(*α*, *β*_1_) and *Y* ~ Ku(*α*, *β*_2_) are independent random variables, then

(4) $$\begin{array}{c} R^{|a,\;b}=\left\{\begin{array}{c} \frac{\beta_{2}}{\beta_{1}+\beta_{2}};\;a=b,\\ \frac{\beta_{2}}{\beta_{1}+\beta_{2}}{\left(\frac{1-b^{\alpha }}{1-a^{\alpha }}\right)}^{\beta_{1}},\;a<b,\;\\ 1-\frac{\beta_{1}}{\beta_{1}+\beta_{2}}{\left(\frac{1-a^{\alpha }}{1-b^{\alpha }}\right)}^{\beta_{2}},\;a>b. \end{array}\right.\; \end{array}$$

**Proof**

Use and substitute functions *f*_*X*_(*x*)=*αβ*_1_*x*^*α*−1^(1 − *x*^*α*^)^*β*_1_−1^, *F*_*X*_(*x*)=1 − (1 − *x*^*α*^)^*β*_1_^, *f*_*Y*_(*y*)=*αβ*_2_*y*^*α*−1^(1 − *y*^*α*^)^*β*_2_−1^, and *F*_*Y*_(*y*)=1 − (1 − *y*^*α*^)^*β*_2_^ in equation (2). So, let *a*=*b*, then

(5) $$\begin{array}{c} R^{|a,b}=\frac{1-F_{Y}\left(a\right)-\int_{a}^{+\infty }F_{X}\left(y\right)f_{Y}\left(y\right)\text{ d}y}{\left(1-F_{X}\left(a\right)\right)\left(1-F_{Y}\left(b\right)\right)}\\ =\frac{1-F_{Y}\left(a\right)-\int_{a}^{1}\left[1-{\left(1-y^{\alpha }\right)}^{\beta_{1}}\right]\alpha \beta_{2}y^{\alpha -1}{\left(1-y^{\alpha }\right)}^{\beta_{2}-1}\text{ d}y}{{\left(1-a^{\alpha }\right)}^{\beta_{1}+\beta_{2}}}\\ =\frac{1-F_{Y}\left(a\right)-\int_{a}^{1}\alpha \beta_{2}y^{\alpha -1}{\left(1-y^{\alpha }\right)}^{\beta_{2}-1}\text{ d}y+\int_{a}^{1}\alpha \beta_{2}y^{\alpha -1}{\left(1-y^{\alpha }\right)}^{\beta_{1}+\beta_{2}-1}\text{ d}y}{{\left(1-a^{\alpha }\right)}^{\beta_{1}+\beta_{2}}}\\ =\;\frac{1-F_{Y}\left(a\right)-\int_{a}^{1}f_{Y}\left(y\right)\text{d}y+\left(\beta_{2}/\left(\beta_{1}+\beta_{2}\right)\right)\int_{a}^{1}\alpha \left(\beta_{1}+\beta_{2}\right)y^{\alpha -1}{\left(1-y^{\alpha }\right)}^{\beta_{1}+\beta_{2}-1}\text{ d}y}{{\left(1-a^{\alpha }\right)}^{\beta_{1}+\beta_{2}}}\\ =\frac{\left(\beta_{2}/\left(\beta_{1}+\beta_{2}\right)\right){\left(1-a^{\alpha }\right)}^{\beta_{1}+\beta_{2}}}{{\left(1-a^{\alpha }\right)}^{\beta_{1}+\beta_{2}}}=\frac{\beta_{2}}{\beta_{1}+\beta_{2}}. \end{array}$$

And, if *a* < *b*, then

(6) $$\begin{array}{c} R^{|a,b}=\frac{1-F_{Y}\left(b\right)-\int_{b}^{+\infty }F_{X}\left(y\right)f_{Y}\left(y\right)\text{ d}y}{\left(1-F_{X}\left(a\right)\right)\left(1-F_{Y}\left(b\right)\right)}\\ =\frac{1-F_{Y}\left(b\right)-\int_{b}^{1}\left[1-{\left(1-y^{\alpha }\right)}^{\beta_{1}}\right]\alpha \beta_{2}y^{\alpha -1}{\left(1-y^{\alpha }\right)}^{\beta_{2}-1}\text{ d}y}{{\left(1-a^{\alpha }\right)}^{\beta_{1}}{\left(1-b^{\alpha }\right)}^{\beta_{2}}}\\ =\frac{\beta_{2}}{\beta_{1}+\beta_{2}}{\left(\frac{1-b^{\alpha }}{1-a^{\alpha }}\right)}^{\beta_{1}}. \end{array}$$

Eventually, if *a* > *b*, we can calculate *R*^*|a*,*b*^ like previous ones.

(7) $$\begin{array}{c} R^{|a,b}=\frac{\int_{a}^{+\infty }F_{Y}\left(x\right)f_{X}\left(x\right)\text{ d}x-F_{Y}\left(b\right)\left(1-F_{X}\left(a\right)\right)}{\left(1-F_{X}\left(a\right)\right)\left(1-F_{Y}\left(b\right)\right)}\\ =\frac{\int_{a}^{1}\left[1-{\left(1-x^{\alpha }\right)}^{\beta_{2}}\right]\alpha \beta_{1}x^{\alpha -1}{\left(1-x^{\alpha }\right)}^{\beta_{1}-1}\text{ d}x-F_{Y}\left(b\right)\left(1-F_{X}\left(a\right)\right)}{{\left(1-a^{\alpha }\right)}^{\beta_{1}}{\left(1-b^{\alpha }\right)}^{\beta_{2}}}\\ =\frac{{\left(1-a^{\alpha }\right)}^{\beta_{1}}{\left(1-b^{\alpha }\right)}^{\beta_{2}}-\left(\beta_{1}/\left(\beta_{1}+\beta_{2}\right)\right){\left(1-a^{\alpha }\right)}^{\beta_{1}+\beta_{2}}}{{\left(1-a^{\alpha }\right)}^{\beta_{1}}{\left(1-b^{\alpha }\right)}^{\beta_{2}}}\\ =1-\frac{\beta_{1}}{\beta_{1}+\beta_{2}}{\left(\frac{1-a^{\alpha }}{1-b^{\alpha }}\right)}^{\beta_{2}}. \end{array}$$

In continuation of this section, we find MLE of *R*^*|a*,*b*^, and then asymptotic distribution of this estimator is found in order to construct its confidence interval.

Let *X*_1_, *X*_2_,…, *X*_*n*_ be a random sample of size *n* of Ku(*α*, *β*_1_) and *Y*_1_, *Y*_2_,…, *Y*_*m*_ be a random sample of size *m* of Ku(*α*, *β*_2_) such that *X* and *Y* are independent. Then, the likelihood function is obtained.

(8) $$\begin{array}{c} L\left(\alpha ,\beta_{1},\beta_{2}\right)=\alpha^{n+m}\beta_{1}^{n}\beta_{2}^{m}\prod_{i=1}^{n}x_{i}^{\alpha -1}\prod_{i=1}^{n}{\left(1-x_{i}^{\alpha }\right)}^{\beta_{1}-1}\times \prod_{j=1}^{m}y_{j}^{\alpha -1}\prod_{j=1}^{m}{\left(1-y_{j}^{\alpha }\right)}^{\beta_{2}-1}. \end{array}$$

Then, to facilitate the calculation, use the form of the likelihood function in [14].

(9) $$\begin{array}{c} l\left(\alpha ,\beta_{1},\beta_{2}\right)=\ln\left[L\left(\alpha ,\beta_{1},\beta_{2}\right)\right]\;=\left(n+m\right)\ln\;\alpha +n\;\ln\;\beta_{1}+m\;\ln\;\beta_{2}+\left(\alpha -1\right)\left(l_{x}+l_{y}\right)+\left(\beta_{1}-1\right)l_{xx}+\left(\beta_{2}-1\right)l_{yy}, \end{array}$$

where

(10) $$\begin{array}{c} l_{x}=\sum_{i=1}^{n}\ln\;x_{i},\\ l_{y}=\sum_{j=1}^{m}\ln\;y_{j},\\ l_{xx}=\sum_{i=1}^{n}\ln\left(1-x_{i}^{\alpha }\right),\\ l_{yy}=\sum_{j=1}^{m}\ln\left(1-y_{j}^{\alpha }\right). \end{array}$$

Therefore, the MLE parameters *α*, *β*_1_, and *β*_2_ are obtained by solving the following equations:

(11) $$\begin{array}{c} \frac{\partial l\left(\alpha ,\beta_{1},\beta_{2}\right)}{\partial \alpha }=0,\\ \frac{\partial l\left(\alpha ,\beta_{1},\beta_{2}\right)}{\partial \beta_{1}}=0,\\ \frac{\partial l\left(\alpha ,\beta_{1},\beta_{2}\right)}{\partial \beta_{2}}=0. \end{array}$$

Then,

(12) $$\begin{array}{c} \hat{\beta_{1}}=-\frac{n}{l_{xx}},\\ \hat{\beta_{2}}=-\frac{m}{l_{yy}}, \end{array}$$

and the MLE parameter *α* is obtained by solving the following nonlinear equation:

(13) $$\begin{array}{c} \frac{n+m}{\alpha }+l_{x}+l_{y}-\hat{\left(\beta_{1}-1\right)}\sum_{i=1}^{n}\frac{x_{i}^{\alpha }\ln\;x_{i}}{1-x_{i}^{\alpha }}-\hat{\left(\beta_{2}-1\right)}\sum_{j=1}^{m}\frac{y_{j}^{\alpha }\ln\;y_{j}}{1-y_{j}^{\alpha }}=0. \end{array}$$

The above equation is solved by numerically methods.

Therefore, the MLE of ${\hat{R}}^{|a,b}$ becomes

(14) $$\begin{array}{c} {\hat{R}}^{|a,b}=\left\{\begin{array}{c} \frac{\hat{\beta_{2}}}{\hat{\beta_{1}}+\hat{\beta_{2}}},\;a=b,\\ \frac{\hat{\beta_{2}}}{\hat{\beta_{1}}+\hat{\beta_{2}}}{\left(\frac{1-b^{\hat{\alpha }}}{1-a^{\hat{\alpha }}}\right)}^{\hat{\beta_{1}}},\;a<b,\;\\ 1-\frac{\hat{\beta_{1}}}{\hat{\beta_{1}}+\hat{\beta_{2}}}{\left(\frac{1-a^{\hat{\alpha }}}{1-b^{\hat{\alpha }}}\right)}^{\hat{\beta_{2}}},\;a>b. \end{array}\right. \end{array}$$

### 2.1. Asymptotic Distribution of ${\hat{R}}^{|a,b}$

In this section, we compute the asymptotic distributions of $\hat{\theta }=\left(\hat{\alpha },\hat{\beta_{1}},\hat{\beta_{2}}\right)$ and ${\hat{R}}^{|a,b}$. Hence, the Fisher information matrix of *θ*=(*α*, *β*_1_, *β*_2_) denoted by *J*(*θ*)=*E*[*I*, *θ*] is given as follows, where **I**=[*I*_*i*,*j*_]_*i*,*j*=1,2,3_ is the observed information matrix, i.e.,

(15) $$\begin{array}{c} I\left(\theta \right)=-\left(\begin{array}{ccc} \frac{\partial^{2}l\left(\alpha ,\beta_{1},\beta_{2}\right)}{\partial \alpha^{2}} & \frac{\partial^{2}l\left(\alpha ,\beta_{1},\beta_{2}\right)}{\partial \alpha \;\partial \beta_{1}} & \frac{\partial^{2}l\left(\alpha ,\beta_{1},\beta_{2}\right)}{\partial \alpha \;\partial \beta_{2}}\\ \frac{\partial^{2}l\left(\alpha ,\beta_{1},\beta_{2}\right)}{\partial \beta_{1}\partial \alpha } & \frac{\partial^{2}l\left(\alpha ,\beta_{1},\beta_{2}\right)}{\partial \beta_{1}{}^{2}} & \frac{\partial^{2}l\left(\alpha ,\beta_{1},\beta_{2}\right)}{\partial \beta_{1}\partial \beta_{2}}\\ \frac{\partial^{2}l\left(\alpha ,\beta_{1},\beta_{2}\right)}{\partial \beta_{2}\partial \alpha } & \frac{\partial^{2}l\left(\alpha ,\beta_{1},\beta_{2}\right)}{\partial \beta_{2}\partial \beta_{1}} & \frac{\partial^{2}l\left(\alpha ,\beta_{1},\beta_{2}\right)}{\partial \beta_{2}{}^{2}} \end{array}\right)=\left(\begin{array}{ccc} I_{1\;1} & I_{1\;2} & I_{1\;3}\\ I_{2\;1} & I_{2\;2} & I_{2\;3}\\ I_{3\;1} & I_{3\;2} & I_{3\;3} \end{array}\right), \end{array}$$

and the elements of *I*(*θ*) are as follows:

(16) $$\begin{array}{c} I_{11}=\frac{n+m}{\alpha^{2}}+\left(\beta_{1}-1\right)\overset{n}{\underset{i=1}{\sum }}\frac{x_{i}^{\alpha }\ln^{2}x_{i}}{{\left(1-x_{i}^{\alpha }\right)}^{2}}+\left(\beta_{2}-1\right)\overset{m}{\underset{j=1}{\sum }}\frac{y_{j}^{\alpha }\ln^{2}y_{j}}{{\left(1-y_{j}^{\alpha }\right)}^{2}},\\ I_{12}=I_{21}=\overset{n}{\underset{i=1}{\sum }}\frac{x_{i}^{\alpha }\ln\;x_{i}}{1-x_{i}^{\alpha }},\\ I_{13}=I_{31}=\overset{m}{\underset{j=1}{\sum }}\frac{y_{j}^{\alpha }\ln\;y_{j}}{1-y_{j}^{\alpha }}I_{22}=\frac{n}{\beta_{1}^{2}},\\ I_{33}=\frac{m}{\beta_{2}^{2}}. \end{array}$$

The elements of the Fisher information matrix are obtained by taking the expectations of the observed matrix. The following integrals can be helpful when finding the elements of the Fisher information matrix:

(17) $$\begin{array}{c} \int_{0}^{1}t^{x-1}\;{\left(1-t\right)}^{y-1}\ln\;t\text{ d}t=B\left(x,y\right)\left(\psi \left(x\right)-\psi \left(x+y\right)\right),\\ \int_{0}^{1}t^{x-1}{\left(1-t\right)}^{y-1}\ln^{2}\;t\text{ d}t=B\left(x,y\right)\left[{\left(\psi \left(x\right)-\psi \left(x+y\right)\right)}^{2}+\psi^{\prime }\left(x\right)-\psi^{\prime }\left(x+y\right)\right], \end{array}$$

where *B*(*x*, *y*) is the beta function, *ψ*(*x*) is the digamma function, and

(18) $$\begin{array}{c} \psi^{\prime }\left(x\right)=\frac{\partial }{\partial x}\psi \left(x\right),\\ J_{11}=\frac{n+m}{\alpha^{2}}+\frac{n\beta_{1}\left(\beta_{1}-1\right)}{\alpha^{2}}B\left(2,\beta_{1}-2\right){\left[\psi \left(2\right)-\psi \left(\beta_{1}\right)\right]}^{2}{\left[\psi^{\prime }\left(2\right)-\psi^{\prime }\left(\beta_{1}\right)\right]}^{2}+\frac{m\beta_{2}\left(\beta_{2}-1\right)}{\alpha^{2}}B\left(2,\beta_{2}-2\right){\left[\psi \left(2\right)-\psi \left(\beta_{2}\right)\right]}^{2}{\left[\psi^{\prime }\left(2\right)-\psi^{\prime }\left(\beta_{2}\right)\right]}^{2},\\ J_{12}=J_{21}=\frac{n\beta_{1}}{\alpha }B\left(2,\beta_{1}-1\right)\left(\psi \left(2\right)-\psi \left(\beta_{1}+1\right)\right),\\ J_{13}=J_{31}=\frac{m\beta_{2}}{\alpha }B\left(2,\beta_{2}-1\right)\left(\psi \left(2\right)-\psi \left(\beta_{2}+1\right)\right),\\ J_{22}=\frac{n}{\beta_{1}^{2}},\\ J_{23}=J_{32}=0,\;\\ J_{33}=\frac{m}{\beta_{2}^{2}}, \end{array}$$

where

(19) $$\begin{array}{c} \psi \left(1\right)=-\gamma ,\\ \psi \left(2\right)=1-\gamma ,\\ \gamma =-\Gamma^{\prime }\left(1\right),\;\\ \psi^{\prime }\left(2\right)=\frac{\pi }{6}-1,\\ \psi^{\prime }\left(\beta_{i}\right)=\sum_{n=0}^{\infty }\frac{1}{{\left(\beta_{i}+n\right)}^{2}},\;i=1,2. \end{array}$$

So, $J\left(\theta \right)=\left(\begin{array}{ccc} J_{11} & J_{12} & J_{13}\\ J_{21} & J_{2\;2} & J_{23}\\ J_{31} & J_{32} & J_{33} \end{array}\right).$ As *n*⟶*∞*  and *m*⟶*∞*, then by using of multivariate CLT of $\hat{\theta }$, we have $\hat{\theta }⟶N_{3}\left(\theta ,\;\Sigma \right)$ where $\hat{\theta }=\left(\;\hat{\alpha },\hat{\beta_{1}},\hat{\beta_{2}}\right)$ and Σ is inverse of the Fisher information matrix:

(20) $$\begin{array}{c} \Sigma =\frac{1}{\det\;J\left(\theta \right)}\left(\begin{array}{ccc} J_{22}J_{33}-J_{23}J_{32} & J_{13}J_{32}-J_{12}J_{33} & J_{12}J_{23}-J_{13}J_{22}\\ J_{23}J_{31}-J_{21}J_{33} & J_{11}J_{33}-J_{13}J_{31} & J_{13}J_{21}-J_{11}J_{23}\\ J_{21}J_{32}-J_{22}J_{31} & J_{12}J_{31}-J_{11}J_{32} & J_{11}J_{22}-J_{\;12}J_{21} \end{array}\right)=\frac{1}{\det\;J\left(\theta \right)}\left(\begin{array}{ccc} J_{22}J_{33} & -J_{12}J_{33} & -J_{13}J_{22}\\ -J_{21}J_{33} & J_{11}J_{33}-J_{13}J_{31} & J_{13}J_{21}\\ -J_{22}J_{31} & J_{12}J_{31} & J_{11}J_{22}-J_{\;12}J_{21} \end{array}\right), \end{array}$$

such that

(21) $$\begin{array}{c} \det J\left(\theta \right)=J_{11}J_{22}J_{33}+J_{12}J_{\;23}J_{31}+J_{13}J_{21}J_{32}-J_{13}J_{22}J_{31}-J_{\;12}J_{21}J_{33}-J_{11}J_{23}J_{32}\\ =J_{11}J_{22}J_{33}-J_{13}J_{22}J_{31}-J_{\;12}J_{21}J_{33}\\ =\frac{nm}{\beta_{1}^{2}\beta_{2}^{2}\alpha^{2}}\left\{n+m+n\beta_{1}\left(\beta_{1}-1\right)B\left(2,\beta_{1}-2\right){\left[\psi \left(2\right)-\psi \left(\beta_{1}\right)\right]}^{2}{\left[\psi^{\prime }\left(2\right)-\psi^{\prime }\left(\beta_{1}\right)\right]}^{2}+m\beta_{2}\left(\beta_{2}-1\right)B\left(2,\beta_{2}-2\right){\left[\psi \left(2\right)-\psi \left(\beta_{2}\right)\right]}^{2}\left[\psi^{\prime }\left(2\right)-\psi^{\prime }\left(\beta_{2}\right)\right]\right\}-\frac{nm^{2}}{\beta_{1}^{2}\alpha^{2}}{\left\{\beta_{2}B\left(2,\beta_{2}-1\right)\left[\psi \left(2\right)-\psi \left(\beta_{2}+1\right)\right]\right\}}^{2}\\ -\frac{mn^{2}}{\beta_{2}^{2}\alpha^{2}}{\left\{\beta_{1}B\left(2,\beta_{1}-1\right)\left[\psi \left(2\right)-\psi \left(\beta_{1}+1\right)\right]\right\}}^{2}. \end{array}$$

Now, we can use the Delta method to express the following theorem.

**Theorem 2 .**

As *n*⟶*∞*  and *m*⟶*∞*, then

(22) $$\begin{array}{c} \left\{\begin{array}{c} \frac{{\hat{R}}^{|a,b}-R^{|a,b}}{\sigma_{1}}⟶N\left(0,1\right);\;a=b,\\ \frac{{\hat{R}}^{|a,b}-R^{|a,b}}{\sigma_{2}}⟶N\left(0,1\right);\;a<b,\\ \frac{{\hat{R}}^{|a,b}-R^{|a,b}}{\sigma_{3}}\;⟶N\left(0,1\right);\;a>b, \end{array}\right. \end{array}$$

where *σ*_1_^2^, *σ*_2_^2^, and *σ*_3_^2^ are obtained as the following equations:

(23) $$\begin{array}{c} \sigma_{1}^{2}=\frac{\beta_{1}^{2}\beta_{2}^{2}}{nm{\left(\beta_{1}+\beta_{2}\right)}^{4}\left(A-C-D\right)}\left\{m\left(A-C\right)-2nm\beta_{1}^{2}\beta_{2}^{2}B_{3}B_{4}\psi_{3}\psi_{4}+n\left(A-D\right)\right\},\\ \sigma_{2}^{2}=\frac{1}{\left(A-C-D\right)}\left\{\rho_{1}^{2}\alpha^{2}+\rho_{2}^{2}\beta_{1}^{2}\left(A-C\right)+\rho_{3}^{2}\beta_{2}^{2}\left(A-D\right)-2\rho_{1}\rho_{2}\alpha \beta_{1}^{3}-2\rho_{1}\rho_{3}\alpha \beta_{2}^{3}+2\rho_{2}\rho_{3}\beta_{1}^{3}\beta_{2}^{3}B_{3}B_{4}\psi_{3}\psi_{4}\right\},\\ \sigma_{3}^{2}=\frac{1}{\left(A-C-D\right)}\left\{\gamma_{1}^{2}\alpha^{2}-2\gamma_{1}\gamma_{2}\alpha \beta_{1}^{3}-2\gamma_{1}\gamma_{3}\alpha \beta_{2}^{3}+2\gamma_{2}\gamma_{3}\beta_{1}^{3}\beta_{2}^{3}B_{3}B_{4}\psi_{3}\psi_{4}+\frac{\gamma_{2}^{2}\beta_{1}^{2}}{n}\left(A-C\right)+\frac{\gamma_{3}^{2}\beta_{2}^{2}}{m}\left(A-D\right)\right\}, \end{array}$$

where

(24) $$\begin{array}{c} B_{1}=B\left(2,\beta_{1}-2\right),\;B_{2}=B\left(2,\beta_{2}-2\right)\;,B_{3}=\;B\left(2,\beta_{2}-1\right),B_{4}=B\left(2,\beta_{1}-1\right),\\ \psi_{1}=\psi \left(2\right)-\psi \left(\beta_{1}\right),\;\psi_{2}=\psi \left(2\right)-\psi \left(\beta_{2}\right),\;\psi_{3}=\psi \left(2\right)-\psi \left(\beta_{2}+1\right),\psi_{4}=\psi \left(2\right)-\psi \left(\beta_{1}+1\right),\\ \psi_{1}^{\prime }=\psi^{\prime }\left(2\right)-\psi^{\prime }\left(\beta_{1}\right),\;\psi_{2}^{\prime }=\psi^{\prime }\left(2\right)-\psi^{\prime }\left(\beta_{2}\right),\\ A=n+m+n\beta_{1}\left(\beta_{1}-1\right)B_{1}\psi_{1}^{2}\psi_{1}^{\prime }+m\beta_{2}\left(\beta_{2}-1\right)B_{2}\psi_{2}^{2}\psi_{2}^{\prime },\\ C=m\beta_{2}^{4}B_{3}^{2}\psi_{3}^{2},D=n\beta_{1}^{4}B_{4}^{2}\psi_{4}^{2},\\ \rho_{1}=\frac{\beta_{1}\beta_{2}}{\beta_{1}+\beta_{2}}{\left(\frac{1-b^{\alpha }}{1-a^{\alpha }}\right)}^{\beta_{1}-1}\frac{a^{\alpha }\left(1-b^{\alpha }\right)\ln\;a-b^{\alpha }\left(1-a^{\alpha }\right)\ln\;b}{{\left(1-a^{\alpha }\right)}^{2}},\\ \rho_{2}=\frac{\beta_{2}}{\beta_{1}+\beta_{2}}{\left(\frac{1-b^{\alpha }}{1-a^{\alpha }}\right)}^{\beta_{1}}\left[\ln\left(\frac{1-b^{\alpha }}{1-a^{\alpha }}\right)-\frac{1}{\beta_{1}+\beta_{2}}\right],\\ \rho_{3}=\frac{\beta_{1}}{{\left(\beta_{1}+\beta_{2}\right)}^{2}}{\left(\frac{1-b^{\alpha }}{1-a^{\alpha }}\right)}^{\beta_{1}},\gamma_{1}=\frac{\beta_{1}\beta_{2}}{\beta_{1}+\beta_{2}}{\left(\frac{1-a^{\alpha }}{1-b^{\alpha }}\right)}^{\beta_{2}-1}\frac{a^{\alpha }\left(1-b^{\alpha }\right)\ln\;a-b^{\alpha }\left(1-a^{\alpha }\right)\ln\;b}{{\left(1-b^{\alpha }\right)}^{2}},\\ \gamma_{2}=\frac{-\beta_{2}}{{\left(\beta_{1}+\beta_{2}\right)}^{2}}{\left(\frac{1-a^{\alpha }}{1-b^{\alpha }}\right)}^{\beta_{2}},\gamma_{3}=\frac{\beta_{1}}{\beta_{1}+\beta_{2}}{\left(\frac{1-a^{\alpha }}{1-b^{\alpha }}\right)}^{\beta_{2}}\left[\frac{1}{\beta_{1}+\beta_{2}}-\ln\left(\frac{1-a^{\alpha }}{1-b^{\alpha }}\right)\right]. \end{array}$$

**Proof**

Let

(25) $$\begin{array}{c} g\left(x_{1},x_{2},x_{3}\right)=\left\{\begin{array}{c} \frac{x_{3}}{x_{2}+x_{3}};\;a=b,\\ \frac{x_{3}}{x_{2}+x_{3}}{\left(\frac{1-b^{x_{1}}}{1-a^{x_{1}}}\right)}^{x_{2}};\;a<b,\;\\ 1-\frac{x_{2}}{x_{2}+x_{3}}{\left(\frac{1-a^{x_{1}}}{1-b^{x_{1}}}\right)}^{x_{3}};\;a>b, \end{array}\right. \end{array}$$

then

(26) $$\begin{array}{c} \frac{\partial g}{\partial x_{1}}=\left\{\begin{array}{c} 0;\;a=b,\\ \frac{x_{3}x_{2}}{x_{2}+x_{3}}{\left(\frac{1-b^{x_{1}}}{1-a^{x_{1}}}\right)}^{x_{2}-1}\frac{a^{x_{1}}\left(1-b^{x_{1}}\right)\ln\;a-b^{x_{1}}\left(1-a^{x_{1}}\right)\ln\;b}{{\left(1-a^{x_{1}}\right)}^{2}};\;a<b,\;\\ \frac{x_{3}x_{2}}{x_{2}+x_{3}}{\left(\frac{1-a^{x_{1}}}{1-b^{x_{1}}}\right)}^{x_{3}-1}\frac{a^{x_{1}}\left(1-b^{x_{1}}\right)\ln\;a-b^{x_{1}}\left(1-a^{x_{1}}\right)\ln\;b}{{\left(1-b^{x_{1}}\right)}^{2}};\;a>b, \end{array}\right.\\ \frac{\partial g}{\partial x_{2}}=\left\{\begin{array}{c} \frac{-x_{3}}{{\left(x_{2}+x_{3}\right)}^{2}};\;a=b,\\ \frac{x_{3}}{x_{2}+x_{3}}{\left(\frac{1-b^{x_{1}}}{1-a^{x_{1}}}\right)}^{x_{2}}\left[\ln\left(\frac{1-b^{x_{1}}}{1-a^{x_{1}}}\right)-\frac{1}{x_{2}+x_{3}}\right];\;a<b,\\ \frac{-x_{3}}{{\left(x_{2}+x_{3}\right)}^{2}}{\left(\frac{1-a^{x_{1}}}{1-b^{x_{1}}}\right)}^{x_{3}};\;a>b, \end{array}\right.\\ \frac{\partial g}{\partial x_{3}}=\left\{\begin{array}{c} \frac{x_{2}}{{\left(x_{2}+x_{3}\right)}^{2}};\;a=b,\\ \frac{x_{2}}{{\left(x_{2}+x_{3}\right)}^{2}}{\left(\frac{1-b^{x_{1}}}{1-a^{x_{1}}}\right)}^{x_{2}};\;a<b,\\ \frac{x_{2}}{x_{2}+x_{3}}{\left(\frac{1-a^{x_{1}}}{1-b^{x_{1}}}\right)}^{x_{3}}\left[\frac{1}{x_{2}+x_{3}}-\ln\left(\frac{1-a^{x_{1}}}{1-b^{x_{1}}}\right)\right];\;a>b, \end{array}\right.\\ \dot{\;g\left(x\right)}=\left(\frac{\partial g\left(x_{1},x_{2},x_{3}\right)}{\partial x_{1}},\frac{\partial g\left(x_{1},x_{2},x_{3}\right)}{\partial x_{2}},\frac{\partial g\left(x_{1},x_{2},x_{3}\right)}{\partial x_{3}}\right). \end{array}$$

Since $\;\left(\hat{\theta }-\theta \right)⟶N\left(0,\;\Sigma \right)$, by Cramer's theorem,

(27) $$\begin{array}{c} \left(g\left(\hat{\theta }\right)-g\left(\theta \right)\right)⟶N\left(\;0,\;\dot{g\left(\theta \right)}\Sigma \dot{g{\left(\theta \right)}^{T}}\right), \end{array}$$

where $\dot{g\left(\theta \right)}=\partial R^{|a,b}/\partial \theta =\left(\partial R^{|a,b}/\partial \alpha ,\partial R^{|a,b}/\partial \beta_{1},\partial R^{|a,b}/\partial \beta_{2}\right)$. Now, *σ*_1_^2^, *σ*_2_^2^, and *σ*_3_^2^ are computed of $\dot{g\left(\theta \right)}\Sigma \dot{g{\left(\theta \right)}^{T}}$ for case of *a*=*b*, *a* < *b*, and *a* > *b*, respectively.

Using the previous theorem, the confidence interval for *R*^*|a*,*b*^ can be obtained.

**Theorem 3 .**

(1 − *α*) 100% confidence interval for *R*^*|a*,*b*^ is equal to

(28) $$\begin{array}{c} \left\{\begin{array}{c} R^{|a,b}\in \left({\hat{R}}^{|a,b}-z_{1-\alpha /2}{\hat{\sigma }}_{1}\;,\;{\hat{R}}^{|a,b}+z_{1-\alpha /2}{\hat{\sigma }}_{1}\right);\;a=b,\\ R^{|a,b}\in \left({\hat{R}}^{|a,b}-z_{1-\alpha /2}{\hat{\sigma }}_{2}\;,\;{\hat{R}}^{|a,b}+z_{1-\alpha /2}{\hat{\sigma }}_{2}\right);\;a<b,\\ R^{|a,b}\in \left({\hat{R}}^{|a,b}-z_{1-\alpha /2}{\hat{\sigma }}_{3}\;,\;{\hat{R}}^{|a,b}+z_{1-\alpha /2}{\hat{\sigma }}_{3}\right);\;a>b. \end{array}\right. \end{array}$$

In above equations ${\hat{\sigma }}_{i}^{2}\text{ where }i=1,2,3$ are similar to *σ*_*i*_^2^ where *i*=1,2,3 in Theorem 2 with substitutions $\;{\hat{\;\alpha }}_{\;}$, ${\hat{\beta }}_{1}$, and ${\hat{\beta }}_{2}$ instead of  *α*, *β*_1_, and  *β*_2_.

### 2.2. Bayesian Estimation of **R**^*| ***a**,**b**^

In this section, we use the Bayesian method to approximate the posterior distribution for the values of interest in the multicomponent stress strength for the Kumarswamy distribution based on the MCMC method.

Therefore, suppose *π*(*θ*)=*π*(*α*)*π*(*β*_1_)*π*(*β*_2_) is the joint prior density for *θ*=(*α*, *β*_1_, *β*_2_), such that *α* ~ Γ(*a*_1_, *b*_1_),  *β*_1_ ~ Γ(*a*_2_, *b*_2_), and *β*_2_ ~ Γ(*a*_3_, *b*_3_).

In these priors, Γ(*a*_*i*_, *b*_*i*_) is the gamma distribution with mean and variance *a*_*i*_/*b*_*i*_ and *a*_*i*_/*b*_*i*_^2^, respectively, such that *a*_*i*_ and *b*_*i*_ are known parameters and  *a*_*i*_, *b*_*i*_ > 0. We assume that the prior distributions *α*, *β*_1_, and  *β*_2_ are independent.

(29) $$\begin{array}{c} \pi \left(\alpha \right)\propto \;\alpha^{a_{1}-1}e^{-\alpha b_{1}},\pi \left(\beta_{1}\right)\propto \;\beta_{1}{}^{a_{2}-1}e^{-\beta_{1}b_{2}},\pi \left(\beta_{2}\right)\propto \;\beta_{2}{}^{a_{3}-1}e^{-\beta_{2}b_{3}}. \end{array}$$

Then, *π*(*θ*, **x**, **y**)=*π*(*α*)*π*(*β*_1_)*π*(*β*_2_)*L*(*α*, *β*_1_, *β*_2_).

(30) $$\begin{array}{c} \propto \alpha^{a_{1}+m+n-1}e^{-\alpha b_{1}}\;\beta_{1}^{a_{2}+n-1}e^{-\beta_{1}b_{2}}\beta_{2}^{a_{3}+m-1}e^{-\beta_{2}b_{3}}\beta_{2}^{m}\times \prod_{i=1}^{n}x_{i}^{\alpha -1}\prod_{i=1}^{n}{\left(1-x_{i}^{\alpha }\right)}^{\beta_{1}-1}\prod_{i=1}^{n}y_{j}^{\alpha -1}\prod_{i=1}^{n}{\left(1-y_{j}^{\alpha }\right)}^{\beta_{2}-1}. \end{array}$$

Hence,

(31) $$\begin{array}{c} \pi \left(\alpha |\beta_{1},\beta_{2},x,y\right)\propto \Gamma \left(a_{1}+m+n,b_{1}\right)\;L\left(\alpha ,\beta_{1},\beta_{2}\right),\\ \pi \left(\beta_{1}|\alpha ,\beta_{2},x,y\right)\propto \Gamma \left(a_{2}+n,b_{2}\right)\prod_{i=1}^{n}{\left(1-x_{i}^{\alpha }\right)}^{\beta_{1}-1},\\ \pi \left(\beta_{2}|\alpha ,\beta_{1},x,y\right)\propto \Gamma \left(a_{3}+m,b_{3}\right)\prod_{j=1}^{m}{\left(1-y_{j}^{\alpha }\right)}^{\beta_{2}-1}. \end{array}$$

Bayesian inference for parameters *α*, *β*_1_, and *β*_2_ can be performed using the Metropolis–Hastings algorithm (see Chib and Greenberg [15]) considering the conditional distributions as the target densities.

### 2.3. Bootstrap Confidence Intervals

Similar to Section 2.2, we study confidence interval based on the percentile bootstrap method for the case of common shape parameter. The algorithm is as follows:

Step 1: For samples {*x*_1_,…,  *x*_*n*_} and {*y*_1_,…,  *y*_*m*_}, calculate $\hat{\;\theta }=\left(\hat{\alpha },\hat{\beta_{1}},\hat{\beta_{2}}\right)$.
Step 2: Use ($\hat{\alpha },\hat{\beta_{1}}$) and ($\hat{\alpha }$, $\hat{\beta_{2}}$) to generate bootstrap samples {*x*_1_^*∗*^,…, *x*_*n*_^*∗*^} and {*y*_1_^*∗*^,…, *y*_*m*_^*∗*^}, respectively, and then using the generated samples, calculate the bootstrap estimate of *R*  say *R*^*∗*^ as follows:
  (32) $$\begin{array}{c} \hat{R^{*}}=\left\{\begin{array}{c} \frac{\hat{\beta_{2}}}{\hat{\beta_{1}}+\hat{\beta_{2}}};\;a=b,\\ \frac{\hat{\beta_{2}}}{\hat{\beta_{1}}+\hat{\beta_{2}}}{\left(\frac{1-b^{\hat{\alpha }}}{1-a^{\hat{\alpha }}}\right)}^{\hat{\beta_{1}}};\;a<b,\;\\ 1-\frac{\hat{\beta_{1}}}{\hat{\beta_{1}}+\hat{\beta_{2}}}{\left(\frac{1-a^{\hat{\alpha }}}{1-b^{\hat{\alpha }}}\right)}^{\hat{\beta_{2}}};\;a>b. \end{array}\right. \end{array}$$
Step 3: Repeat the previous step with time N bootstrap to generate ${\hat{\;R}}_{1}^{*},\ldots ,{\hat{R}}_{N}^{*}$.

Now, ${\hat{R}}_{\text{boot}}^{*}=1/N\sum_{i=1}^{N}{\hat{R}}_{i}^{*}$ and the approximate 100 (1 − *α*) % confidence interval of *R*_ _^ *G*^ is given by (${\hat{R}}_{\left(\alpha /2\right)}^{*},{\hat{R}}_{\left(1-\alpha /2\right)}^{*}$) where ${\hat{R}}_{\left(\gamma \right)}^{*}$ shows quantile of order *γ* for ${\hat{\;R}}_{1}^{*},\ldots ,{\hat{R}}_{N}^{*}$.

## 3. Simulation Study

In this section, we want to simulate the results for different sample sizes to better illustrate the methods presented in this paper (classical estimation, Bayesian estimation, and bootstrap). Codes of this section have been provided in Appendix. The two known parameters *a*=0.1 and *b*=0.2 are used in our study. For other parameters in the model, we consider two cases *α*=5, *β*_1_=3.5, and *β*_2_=3.25 which lead to *R*^*|a*,*b*^=0.480959271 and *α*=1.25, *β*_1_=7, and *β*_2_=4.2 that their corresponding *R*^*|a*,*b*^ is  0.20582159557. In this simulation, sample sizes (*n*, *m*)=(5,5),  (10,10), (15,15), (20,20), (35,35), (50,50), (70,70), (100,100), (5,15), (25,15), (20,35), (30,60), (100,55) are used. All results are the mean of 5000 iteration. Estimation has been accomplished by three methods such as MLE, Bayesian, and bootstrap. For comparing these methods, we compute bias and MSE. In addition, coverage probabilities (CPs) and length of confidence interval have been computed. Our findings are represented in Tables 1–6. As these tables demonstrate, Bayesian estimation is the best method among three used methods with respect to criteria MSE, CP, and length of confidence interval. However, ML estimation has less bias than two other studied methods.

## 4. Discussion and Conclusion

The conditional stress-strength model as an interesting extension of the stress-strength model in reliability was studied for Kumaraswamy distribution. Three estimation methods are applied for statistical inference in this model.

The stress-strength model has been investigated for many distributions such as generalized logistic distribution, generalized failure rate distribution, Rayleigh, and half-normal distribution (see, for instance, Rasekhi et al. [16] and Alamri et al. [17]). They may be applied for the conditional stress-strength model, too. We are going to study this model for generalized logistic distribution in the next step.

The generalized stress-strength parameter (*R*^*G*^) introduced by Saber et al. [18] is given by

(33) $$\begin{array}{c} R^{G}=P\left(Y<\;X<Z\right). \end{array}$$

This quantity is related to a system with three components while *R* is defined for systems which have two components.

For future studies, a possible extension of (33) is recommended in the following:

(34) $$\begin{array}{c} R^{G|a,b,c}=P\left(Y<\;X<Z|X>a,Y>b,Z>c\right). \end{array}$$

## Figures and Tables

**Table 1:** Classical estimation: *α*=5, *β*_1_=3.5, *β*_2_=3.25, *a*=0.1, *b*=0.2, and *R*^*|a*,*b*^=0.480959271 for equal sample sizes *n* and *m*.

(*m*, *n*)	${\hat{R}}^{|a,b}$	Bias (${\hat{R}}^{|a,b}$)	MSE (${\hat{R}}^{|a,b}$)	C.I	CP	Length
(5, 5)	0.4842	0.0033	0.0320	(0.2225, 0.7459)	0.8000	0.5235
(10, 10)	0.5135	0.0307	0.0097	(0.3040, 0.7228)	0.9500	0.4188
(15, 15)	0.4833	0.0005	0.0082	(0.3115, 0.6549)	0.9487	0.3433
(20, 20)	0.4754	−0.0068	0.0050	(0.3248, 0.6259)	0.9600	0.3012
(35, 35)	0.4778	−0.0032	0.0028	(0.3625,0.5930)	0.9630	0.2305
(50, 50)	0.4809	0.0000	0.0030	(0.3846, 0.5772)	0.9300	0.1927
(70, 70)	0.48589	0.00493	0.00178	(0.4038, 0.5679)	0.93000	0.16402
(100, 100)	0.4786	−0.0023	0.0010	(0.4098, 0.5474)	0.9700	0.1376

**Table 2:** Bayesian and bootstrap estimations: *α*=5, *β*_1_=3.5, *β*_2_=3.25, *a*=0.1, *b*=0.2, and *R*^*|a*,*b*^=0.480959271 for equal sample sizes *n* and *m*.

(*n*, *m*)	${\hat{R}}_{\text{bayes}}^{|a,b}$	Bias (${\hat{R}}_{\text{bayes}}^{|a,b}$)	MSE (${\hat{R}}_{\text{bayes}}^{|a,b}$)	C.I_bayes_	CP_bayes_	Length_bayes_	C.I_boot_	CP_boot_	Length_boot_
(5, 5)	0.4929	0.0120	0.003	(0.4188, 0.5497)	1	0.1310	(0.1228, 0.8455)	1	0.7227
(10, 10)	0.4967	0.0157	0.0003	(0.4363, 0.5498)	1	0.1135	(0.2709, 0.7559)	1	0.4850
(15, 15)	0.4975	0.0166	0.0003	(0.4412, 0.5498)	1	0.1086	(0.2905, 0.6759)	1	0.3853
(20, 20)	0.4985	0.0175	0.0003	(0.4446, 0.5498)	1	0.1052	(0.3092, 0.6415)	1	0.3323
(35, 35)	0.4989	0.0180	0.0003	(0.4469, 0.5498)	1	0.1030	(0.3538, 0.6017)	1	0.2479
(50, 50)	0.4990	0.0181	0.0003	(0.4475, 0.5498)	1	0.1023	(0.3772, 0.5846)	1	0.2074
(70, 70)	0.49467	0.01371	0.00088	(0.44507, 0.54427)	1	0.09920	(0.40004, 0.5717)	1	0.17169
(100, 100)	0.0190	0.5000	0.0004	(0.4499, 0.5499)	1	0.1000	(0.4138, 0.5434)	1	0.1297

**Table 3:** Classical estimation: *α*=5, *β*_1_=3.5, *β*_2_=3.25, *a*=0.1, *b*=0.2, and *R*^*|a*,*b*^=0.480959271 for different sample sizes *n* and *m*.

(*n*, *m*)	${\hat{R}}^{|a,b}$	Bias (${\hat{R}}^{|a,b}$)	MSE (${\hat{R}}^{|a,b}$)	C.I	CP	Length
(5, 15)	0.4040	−0.1134	0.0413	(0.2015, 0.6065)	0.5000	0.4050
(20, 15)	0.4787	−0.0022	0.0065	(0.3243, 0.6330)	0.9315	0.3087
(20, 35)	0.5007	0.0217	0.0047	(0.3661, 0.6352)	0.8974	0.2691
(30, 60)	0.4755	−0.0055	0.0034	(0.3680, 0.5829)	0.8900	0.2149
(100, 55)	0.4820	0.0010	0.0015	(0.4004, 0.5634)	0.9600	0.1630

**Table 4:** Bayesian and bootstrap Estimations: *α*=5, *β*_1_=3.5, *β*_2_=3.25, *a*=0.1, *b*=0.2, and *R*^*|a*,*b*^=0.480959271 for different sample sizes *n* and *m*.

(*n*, *m*)	${\hat{R}}_{\text{bayes}}^{|a,b}$	Bias (${\hat{R}}_{\text{bayes}}^{|a,b}$)	MSE (${\hat{R}}_{\text{bayes}}^{|a,b}$)	C.I_bayes_	CP_bayes_	Length_bayes_	C.I_boot_	CP_boot_	Length_boot_
(5, 15)	0.4969	0.0159	0.0003	(0.4361, 0.5498)	1.0000	0.1137	(0.1341, 0.6738)	1.0000	0.5398
(20, 15)	0.4981	0.0171	0.0003	(0.4437, 0.54981)	1.000	0.1061	(0.3076, 0.6497)	1.0000	0.3421
(20, 35)	0.4986	0.0177	0.0003	(0.4453, 0.5498)	1.0000	0.1045	(0.3539, 0.6474)	1.0000	0.2935
(30, 60)	0.4990	0.0181	0.0003	(0.4473, 0.5498)	1.0000	0.1025	(0.3598, 0.5911)	1.0000	0.2313
(100, 55)	0.5000	0.0190	0.0004	(0.4499, 0.5499)	1.0000	0.1000	(0.3955, 0.5683)	1.0000	0.1728

**Table 5:** Classical estimation: *α*=1.25, *β*_1_=7, *β*_2_=4.2, *a*=0.1,  *b*=0.2, and *R*_ _^*|a*,*b*^=0.20582159557.

(*n*, *m*)	${\hat{R}}^{|a,b}$	Bias (${\hat{R}}^{|a,b}$)	MSE (${\hat{R}}^{|a,b}$)	C.I	CP	Length
(5, 5)	0.1962	−0.0165	0.0138	(0.0169, 0.4094)	0.7895	0.4265
(25, 25)	0.2148	0.0090	0.0036	(0.1055, 0.3239)	0.9300	0.2184
(30, 30)	0.2009	−0.0049	0.0028	(0.1043, 0.2975)	0.8800	0.1932
(40, 40)	0.2024	−0.0034	0.0017	(0.1180, 0.2868)	0.9500	0.1689

**Table 6:** Bayesian and bootstrap estimations: *α*=1.25, *β*_1_=7, *β*_2_=4.2, *a*=0.1,  *b*=0.2, and *R*_ _^*|a*,*b*^=0.20582159557.

(*n*, *m*)	${\hat{R}}_{\text{bayes}}^{|a,b}$	Bias (${\hat{R}}_{\text{bayes}}^{|a,b}$)	MSE (${\hat{R}}_{\text{bayes}}^{|a,b}$)	C.I_bayes_	CP_bayes_	Length_bayes_	C.I_boot_	CP_boot_	Length_boot_
(5, 5)	0.3462	0.1404	0.0233	(0.1438, 0.5486)	0.9100	0.3048	(-0.0571, 0.4496)	1.0000	0.5069
(25, 25)	0.3055	0.0997	0.0119	(0.1337, 0.4772)	1.0000	0.2435	(0.0974, 0.3320)	1.0000	0.2346
(30, 30)	0.3035	0.0977	0.0115	(0.1297, 0.4773)	1.0000	0.2477	(0.0933, 0.3085)	1.0000	0.2152
(40, 40)	0.3001	0.0942	0.0110	(0.1231, 0.4769)	1.0000	0.2538	(0.1093, 0.2955)	1.0000	0.1863

## Data Availability

The data used to support this study are included within the article.

## Appendix

### Program Codes

  rm(list = ls())
*a*<−0.1; *b* < −0.2; *n* < −50; *m* < −50; alfa < −5; beta1<−3.5; beta2<−3.25; *N* < −1000; *n*.boot < −200
  ALFA < −0.05; sigma2a < −0.36; sigma2*b* < −4; *a*1<−1; *b*1<−0.5; *a*2<−1.5; *b*2<−1; *a*3<−1.5; *b*3<−1.25
  alfas < 0.9; beta1s < −6.25; beta2s < -10.25; seed < −32101456; n.mh < −1; n.gs < −10000
  Rab < -function(b1,b2,alf) {
  if(*a*<*b*) *m*1<−(b2/(*b*1 + *b*2))^*∗*^((1 − *b*^alf)/(1 − *a*^alf))^*b*1
  if(*a*>*b*) *m*1<-1-(b1/(*b*1 + *b*2))^*∗*^((1 − *a*^alf)/(1 − *b*^alf))^*b*2
  return(m1) }
  rkus < -function(*n*,alf,bet) {
*u* < -runif(*n*); *d* < -(1 − *u*)^(1/bet); dd < -(1 − *d*)^(1/alf)
  return(dd) }
  lalfa < -function(alf){
  lx < -sum(log(*x*)); ly < -sum(log(*y*)); lxx < -sum(log(1-*x*^alf)); lyy < -sum(log(1-*y*^alf))
  lxxx < -sum(*x*^alf^*∗*^log(*x*)/(1-x^alf)); lyyy < -sum(y^alf^*∗*^log(y)/(1-y^alf))
*d* < -(*n *+ *m*)/alf + lx + ly; *d*1* *<* *-(n/lxx* *+* *1)^*∗*^lxxx; *d*2<-(m/lyy* *+* *1)^*∗*^lyyy; dd < -d + *d*1 + *d*2
  return(dd) }
  thetahat < -function(*n*, *m*,*b*1,*b*2,alf){
*x* < -rkus(n,alf,b1); *y* < -rkus(*m*,alf,*b*2)
  lalfa < -function(alf){
  lx < -sum(log(*x*)); ly < -sum(log(*y*)); lxx < -sum(log(1 − *x*^alf)); lyy < -sum(log(1-*y*^alf))
  lxxx < -sum(*x*^alf^*∗*^log(*x*)/(1 − *x*^alf)); lyyy < -sum(*y*^alf^*∗*^log(*y*)/(1-*y*^alf))
*d* < -(*n* + *m*)/alf + lx + ly; *d*1<-(*n*/lxx+1)^*∗*^lxxx; *d*2<-(*m*/lyy + 1)^*∗*^lyyy; dd < -*d* + *d*1 + *d*2
  return(dd) }
  alfahat < -uniroot(lalfa,c(0.0001, 20))$root
  lxx < -sum(log(1 − *x*^alfahat)); lyy < -sum(log(1 − *y*^alfahat)); beta1hat < --n/lxx; beta2hat < --m/lyy
  return(*c*(beta1hat,beta2hat,alfahat))}
  fishermi < -function(*n*,*m*,*b*1,*b*2,alf){
*a*1<-beta(2, *b*1-2); *a*2<-beta(2, *b*2-2); *a*3<-beta(2, *b*1-1); *a*4<-beta(2, *b*2-1)
*c*1 < -digamma(2)-digamma(*b*1); *c*2 < -digamma(2)-digamma(b2); c11 < -trigamma(2)-trigamma(*b*1); c21 < -trigamma(2)-trigamma(*b*2); *c*3 < -digamma(2)-digamma(*b*1 + 1); *c*4<-digamma(2)-digamma(*b*2 + 1)
*J* < -matrix(0, 3, 3); J[1, 1]<-(*n*+ *m* + *n*^*∗*^b1^*∗*^(b1-1)^*∗*^a1^*∗*^c1^2^*∗*^c11+*m*^*∗*^b2^*∗*^(b2-1)^*∗*^a2^*∗*^c2^*∗*^c21)/alf^2
  J[1, 2] < -J[2, 1] < -*n*b1^*∗*^a3^*∗*^c3/alf; J[1, 3] < -J[3, 1] < -*m*^*∗*^b2^*∗*^a4^*∗*^c4/alf; J[2, 2] < -*n*/*b*1^2; J[3, 3]<-*m*/b2^2
  return(J)}
  moshtag < -function(*b*1, *b*2,alf){
  if(*a*<*b*){ *e*1<--((*b*2/(*b*1 + *b*2)) (((1-*b*^alf)/(1-*a*^alf))^(*b*1-1) (b1 ^*∗*^ (b^alf ^*∗*^ log(b)/(1 - a^alf) - (1 - b^alf) ^*∗*^ (a^alf ^*∗*^ log(a))/(1-a^alf)^2))))
*e*2 < -(*b*2/(*b*1 + *b*2)) ^*∗*^ (((1 − *b*^alf)/(1-*a*^alf))^*b*1 ^*∗*^ log(((1 − *b*^alf)/(1 − a^alf))))-*b*2/(*b*1 + *b*2)^2 ^*∗*^ ((1 − b^alf)/(1 − a^alf))^b1
*e*3<-(1/(*b*1 + *b*2) − b2/(*b*1 + *b*2)^2) ^*∗*^ ((1 - b^alf)/(1 − a^alf))^b1 }
  if(*a*>*b*){
*e*1<-(b1/(*b*1 + *b*2)) ^*∗*^ (((1 − a^alf)/(1 − b^alf))^(b2 − 1) ^*∗*^ (b2 ^*∗*^ (a^alf ^*∗*^ log(a)/(1 − b^alf) − (1 − a^alf) ^*∗*^ (b^alf ^*∗*^ log(b))/(1 − b^alf)^2)))
*e*2<--((1/(*b*1 + *b*2) − b1/(*b*1 + *b*2)^2) ^*∗*^ ((1 − a^alf)/(1 − b^alf))^b2)
*e*3<--((b1/(*b*1 + *b*2)) ^*∗*^ (((1 − a^alf)/(1 − b^alf))^b2 ^*∗*^ log(((1 − a^alf)/(1 - b^alf)))) - b1/(*b*1 + *b*2)^2 ^*∗*^ ((1 a^alf)/(1 - b^alf))^b2) }
  return(*c*(e1,e2,e3))}
  sigma2<-function(*n*,*m*,b1,b2,alf){
  JJ < -fishermi(n,m,b1,b2,alf); mosh < -moshtag(b1,b2,alf); *a*5<-t(mosh)%^*∗*^%solve(JJ)%^*∗*^%mosh
  return(a5) }
  R120 < -Rab(beta1, beta2, alfa)
  R12h < -bias12 < - MSE12 < - L12 < - CP12 < -rep(0, 0); za < -qnorm(1-ALFA/2)
  for(i in 1:N) {
*x* < -rkus(n,alfa, beta1); *y* < -rkus(m,alfa, beta2); tethat < -thetahat(*n*, *m*, beta1, beta2, alfa)
  b1hat < -tethat[1]; b2hat < -tethat[2]; ahat < -tethat[3]
  if((b1hat>2)&(b2hat>2))
  R12hat < -Rab(b1hat,b2hat,ahat); var12 < -sigma2(*n*, *m*, b1hat, b2hat, ahat); bias12[*i*]<-R12hat-R120
  MSE12[*i*] < -(R12hat-R120)^2; L12[*i*] < -2^*∗*^1.96^*∗*^sqrt(var12)
  CP12[*i*] < -sum((R120≥(R12hat-za^*∗*^sqrt(var12))) & (R120≤(R12hat + za^*∗*^sqrt(var12))))
  R12h[i]<-R12hat }
  Rhat < -mean(R12h); bias < -mean(bias12); mse < -mean(MSE12); lm < -mean(L12); cp < -mean(CP12)
  lower < -max(round(N^*∗*^ALFA/2),1); upper < -min(round(N^*∗*^(1-ALFA/2)), *N*)
  fboot < -function(*N*){
  R12bo < -rep(0, 0)
  for(i in 1 : N) {
*x* < -rkus(*n*, alfa, beta1); *y* < -rkus(m,alfa, beta2); tethat < -thetahat(*n*, *m*, beta1, beta2, alfa)
  b1hat < -tethat[1]; b2hat < -tethat[2]; ahat < -tethat[3]
  if((b1hat > 2)&(b2hat > 2))
  R12hat < -Rab(b1hat, b2hat, ahat); R12bo[i]<-R12hat}; Rs < -sort(R12h); lb < -Rs[lower]; ub < -Rs[upper]
*L* < --(lb-ub); CP < -sum((R120 ≥ lb)&(R120 ≤ ub)); dd < -c(L,CP)
  return(dd)}
  Lboot < -c(0,0); CPboot < -rep(0, 0)
  for(i in 1:n.boot) {
  results < -fboot(*N*); Lboot[*i*] < -results[1]; CPboot[*i*]<-results[2]}
  Lboot < -mean(Lboot); CPboot < -mean(CPboot); initial < -c(alfa, beta1,beta2,n,m,R120);
  dmle < -c(Rhat, bias,mse,cp,lm); dboot < -c(CPboot, Lboot); dclassic < -c(initial, dmle,dboot)
  dkus < -function(x,alfa, beta) alfa^*∗*^beta^*∗*^x^(alfa-1)^*∗*^(1-x^alfa)^(beta-1)
  falfa < -function(alfa, beta1,beta2,x,y){
*n* < -length(*x*); *m* < -length(y); *d*1 < -(prod(*x*)^*∗*^prod(*y*))^(alfa − 1); *d*2<-prod(1 − x^alfa)^(beta1 − 1)
*d*3<-prod(1 − *y*^alfa)^(beta2 − 1); *d*4<-dgamma(alfa,*a*1 + *m* + *n*,*b*1); *d*5<-d1^*∗*^d2^*∗*^d3^*∗*^d4
  return(d5)}
  fbeta < -function(beta, alfa,x,a,b){
*n* < -length(*x*); *d*1<-prod(1-x^alfa)^(beta-1); *d*2 < -dgamma(beta,*a* + *n*,b); *d*3<-d1^*∗*^d2
  return(d3)}
  mhalfa < -function(nmh,alfas, beta1s,beta2s,x,y){
  set.seed < -seed; sample < -rep(0,nmh)
  for(i in 1:nmh){
  alfa < -rnorm(1,alfas, sqrt(sigma2a)); a.*p*1 < -falfa(alfa, beta1s,beta2s, *x*, y)
  a.*p*2 < -falfa(alfas, beta1s,beta2s, *x*, *y*); accept.prob < -a.p1/a.p2
  if(is.nan(accept.prob) = = T) sample[*i*] < -alfas
  else {
  ratio < -min(1,accept.prob); *u* < -runif(1)
  if(*u* ≤ ratio) sample[*i*] < -alfa
  else sample[*i*] < -alfas }
  alfas < -sample[*i*] }
  return(mean(sample)) }
  mhbeta < -function(nmh,betas, alfas,x,a,b){
  set.seed < -seed; sample < -rep(0,0)
  for(i in 1:nmh){
  beta < -rnorm(1,betas, sqrt(sigma2b)); a.*p*1<-fbeta(beta, alfas,x,a,b); a.*p*2<-fbeta(betas, alfas,*x*,*a*,*b*)
  accept.prob < -a.*p*1/a.*p*2
  if(is.nan(accept.prob) = = T) sample[i]<-betas
  else {
  ratio < -min(1,accept.prob)
*u* < -runif(1)
  if(*u* ≤ ratio) sample[*i*]<-beta
  else sample[*i*] < -betas }
  betas < -sample[i] }
  return(mean(sample)) }
  GSnew < -function(N.GS,N.MH,alfas, beta1s,beta2s,x,y){
*n* < -length(*x*); *m* < -length(*y*); alfa < -beta1 < -beta2 < -Rhat < -rep(0,N.GS)
  for(i in 1:N.GS){
  alfa[*i*] < -mhalfa(N.MH,alfas, beta1s,beta2s,*x*,*y*); beta1[*i*]<-mhbeta(N.MH,beta1s,alfa[*i*],*x*,*a*2,*b*2) beta2[*i*]<-mhbeta(N.MH,beta2s,alfa[i],*y*, *a*3,*b*3); alfas < -alfa[*i*]; beta1s < -beta1[*i*]; beta2s < -beta2[*i*] Rhat[*i*]<-Rab(beta1[*i*],beta1[*i*],alfa[*i*])}
  R12h < -mean(Rhat); var12<-var(Rhat); bias12<-mean(Rhat-R120); MSE12<-mean((Rhat-R120)^2)
  L12<-2^*∗*^1.96^*∗*^sqrt(var12); CP12<-sum((R120 ≥ (R12h-L12/2)) & (R120≤(R12h + L12/2)))
  R12h1<-sort(Rhat); lownumber < -max(round(N.GS,^*∗*^ALFA/2)1)
  upnumber < -min(round(N.GS^*∗*^(1-ALFA/2)),N.GS); c.*l* < -R12h1[lownumber]; c.*u* < -R12h1[upnumber]
  L12n < -c.u-c.l; CP12n<-sum((R120 ≥ *c*.l) & (R120 ≤ *c*.u));
  dd < -c(R12h, var12, bias12, MSE12, L12, CP12, L12n, CP12n)
  return(dd) }
  R1h < -bias1 < - MSE1 < - *L*1 < -*L*1n < - CP1<-CP1n < -rep(0,0)
  for(i in 1:N) {
*x* < -rkus(n,alfa, beta1); *y* < -rkus(*m*,alfa, beta2)
  resu < -GSnew(n.gs,n.mh,alfas, beta1s,beta2s,x,y)
  R1h[*i*] < -resu[1]; bias1[*i*] < -resu[3]; MSE1[*i*] < -resu[4]; L1[*i*] < -resu[5]
  L1n[*i*] < -resu[7]; CP1[*i*] < -resu[6]; CP1n[*i*] < -resu[8] }
  dbayes < -c(mean(R1h), mean(bias1), mean(MSE1), mean(L1), mean(CP1), mean(L1n), mean(CP1n))

## References

[1] Kotz S., Lumelskii Y., Pensky M. (2003). *The Stress-Strength Model and its Generalization: Theory and Applications*.

[2] Rezaei S., Tahmasbi R., Mahmoodi M. (2010). Estimation of *P*[*Y* < *X*] for generalized Pareto distribution. *Journal of Statistical Planning and Inference*.

[3] Saber M. M., Khorshidian K. (2021). Introduction to reliability for conditional stress-strength parameter. *Journal of Sciences*.

[4] Kumaraswamy P. (1976). Sinepower probability density function. *Journal of Hydrology*.

[5] Kumaraswamy P. (1978). Extended sinepower probability density function. *Journal of Hydrology*.

[6] Sundar V., Subbiah K. (1989). Application of double bounded probability density function for analysis of ocean waves. *Ocean Engineering*.

[7] Fletcher S. G., Ponnambalam K. (1996). Estimation of reservoir yield and storage distribution using moments analysis. *Journal of Hydrology*.

[8] Seifi A., Ponnambalam K., Vlach J. (2000). Maximization of manufacturing yield of systems with arbitrary distributions of component values. *Annals of Operations Research*.

[9] Ponnambalam K., Seifi A., Vlach J. (2001). Probabilistic design of systems with general distributions of parameters. *International Journal of Circuit Theory and Applications*.

[10] Nadar M., Kızılaslan F., Papadopoulos A. (2014). Classical and Bayesian estimation of P(Y<X) for Kumaraswamy’s distribution. *Journal of Statistical Computation and Simulation*.

[11] Nadar M., Kızılaslan F. (2014). Classical and Bayesian estimation of P(X<Y) using upper record values from Kumaraswamy’s distribution. *Statistical Papers*.

[12] Dey S., Mazucheli J., Anis M. Z. (2016). Estimation of reliability of multicomponent stress-strength for a Kumaraswamy distribution. *Communications in Statistics-Theory and Methods*.

[13] Kohansal A. (2019). On estimation of reliability in a multicomponent stress-strength model for a Kumaraswamy distribution based on progressively censored sample. *Statistical Papers*.

[14] Hogg R. V., McKean J. W., Craig A. T. (2012). *Introduction to Mathematical Statistics*.

[15] Chib S., Greenbreg E. (1955). Understanding the metropolis-hastings algorithm. *The American Statistician*.

[16] Rasekhi M., Saber M. M., Yousof H. M. (2020). Bayesian and classical inference of reliability in multicomponent stress-strength under the generalized logistic model. *Communications in Statistics-Theory and Methods*.

[17] Alamri O. A., El-Raouf M. M. A., Ismail E. A. (2021). Estimate stress-strength reliability model using Rayleigh and half-normal distribution. *Computational Intelligence and Neuroscience*.

[18] Saber M. M., Rasekhi M., Yousof H. M. (2021). Generalized stress-strength and generalized multicomponent stress-strength models. *Statistics, Optimization and Information Computing*.

